# Supplementary material for: Analysis of Multiple Risk Factors for Seronegative Rate of Anti-Tick-Borne Encephalitis Virus Immunization in Human Serum
Source: Medicina (Kaunas). 2020 May 20;56(5):244. doi: 10.3390/medicina56050244 (PMC7279439; doi:10.3390/medicina56050244)
Supplement: Supplementary file 1 [file medicina-56-00244-s001.pdf]

Supplementary materials

Table 1. Characteristics of vaccinations status all and seronegative study groups regarding to sex

Basic characteristics	Male<br>n (% all study group)	Female<br>n (% all study group)	Waning immunity Male<br>n (% all study group)	Waning immunity Female<br>n (% all study group)
n (%)	2031 (87.7)	284 (12.3)	281 (12.1)	38 (1.6)
Primary immunization, n (%)	533 (23.0)	93 (4.0)	136 (5.9)	16 (0.7)
Primary immunization and 1 periodic booster, n (%)	865 (37.4)	126 (5.4)	101 (4.4)	17 (0.7)
Primary immunization and 2 periodic booster, n (%)	372 (16.1)	36 (1.6)	27 (1.2)	3 (0.1)
Primary immunization and 3 periodic booster, n (%)	138 (10.0)	17 (0.7)	8 (0.3)	2 (0.1)
Primary immunization and ≥4 periodic booster, n (%)	123 (5.3)	12 (0.5)	9 (0.4)	0 (0.0)

Table 2. Changes of anti-TBEV titer in participants subjected to complete primary vaccination depending on the time of last vaccine dose and sex.

Anti-TBEV titer (range, VIEU/ml)	Time since the vaccination (years)									
	time period complies with the vaccination protocol before first booster immunization								time period beyond the vaccination protocol	
	<1		1		2		3		≥4	
	Male n =87	Female n=25	Male n=101	Female n=14	Male n=201	Female n=35	Male n=55	Female n=9	Male n=89	Female n=10
	n (%)	n (%)	n (%)	n (%)	n (%)	n (%)	n (%)	n (%)	n (%)	n (%)
<120	11 (12.6)	1 (4.0)	20 (19.8)	2 (14.3)	43 (21.4)	6 (17.1)	13 (23.6)	2 (22.2)	22 (24.7)	2 (20.0)
120-164	2 (2.3)	2 (8.0)	4 (4.0)	0 (0.0)	13 (6.5)	1 (2.9)	5 (9.1)	0 (0.0)	3 (3.4)	0 (0.0)
≥ 165	74 (85.1)	22 (88.0)	77 (76.2)	12 (85.7)	145 (72.1)	28 (80.0)	37 (67.3)	7 (77.8)	64 (71.9)	8 (80.0)

Table 3. Changes of anti-TBEV titer in participants subjected to complete primary and booster vaccination depending on the number of doses and sex.

	Primary immunization, n (%)		Primary immunization and 1 periodic booster, n (%)		Primary immunization and 2 periodic booster, n (%)		Primary immunization and 3 periodic booster, n (%)		Primary immunization and ≥4 periodic booster, n (%)	
Anti-TBEV titer (range, VIEU/ml)	Male n =533	Female n=93	Male n=865	Female n=126	Male n=372	Female n=36	Male n=138	Female n=17	Male n=123	Female n=12
	n (%)	n (%)	n (%)	n (%)	n (%)	n (%)	n (%)	n (%)	n (%)	n (%)
<120	109 (20.4)	13 (14.0)	64 (7.4)	14 (11.1)	14 (3.8)	3 (8.3)	6 (4.3)	2 (11.8)	7 (5.7)	0 (0.0)
120-164	27 (5.1)	3 (3.2)	37 (4.3)	3 (2.4)	13 (3.5)	0 (0.0)	2 (1.5)	0 (0.0)	2 (1.6)	0 (0.0)
≥ 165	397 (74.5)	77 (82.8)	764 (88.3)	109 (86.5)	345 (92.7)	33 (91.7)	130 (94.2)	15 (88.2)	114 (92.7)	12 (100.0)

Table 4. Changes of anti-TBEV titer in participants subjected to complete primary vaccination and at least one booster dose regarding to sex.

Age of participants (in years)	Time since the vaccination (in years)	Sex	Antibodies titer in serum (range, VIEU/ml)		
			<120<br>n (%)	120-164<br>n (%)	165-1000<br>n (%)
< 60	<1<br>Total n=401	Male<br>n=339	15 (4.4)	7 (2.1)	317 (93.5)
		Female<br>n=62	7 (11.3)	1 (1.6)	54 (87.1)
	1<br>Total n=321	Male<br>n=284	15 (5.3)	5 (1.8)	264 (92.9)
		Female<br>n=37	2 (5.4)	1 (2.7)	34 (91.9)
	2<br>Total n=480	Male<br>n=419	35 (8.4)	24 (5.7)	360 (85.9)
		Female<br>n=61	7 (11.5)	1 (1.6)	53 (86.9)
	3<br>Total n=156	Male<br>n=141	6 (4.2)	7 (5.0)	128 (90.8)
		Female<br>n=15	0 (0.0)	0 (0.0)	15 (100.0)
	>4<br>Total n=133	Male<br>n=124	8 (6.5)	3 (2.4)	113 (91.1)
		Female<br>n=9	1 (11.1)	0 (0.0)	8 988.9)
≥ 60	<1<br>Total n=50	Male<br>n=47	4 (8.5)	0 (0.0)	43 (91.5)
		Female<br>n=3	1 (33.3)	0 (0.0)	2 (66.7)
	1<br>Total n=43	Male<br>n=42	2 (4.8)	2 (4.8)	38 (90.4)
		Female<br>n=1	0 (0.0)	0 (0.0)	1 (100.0)
	2<br>Total n=63	Male<br>n=61	2 (3.3)	4 (6.6)	55 (90.1)
		Female<br>n=2	1 (50.0)	0 (0.0)	1 (50.0)
	3<br>Total n=21	Male<br>n=20	1 (5.0)	2 (10.0)	17 (85.0)
		Female<br>n=1	0 (0.0)	0 (0.0)	1 (100.0)
	≥4<br>Total n=21	Male<br>n=21	3 (14.3)	0 (0.0)	18 (85.7)
		Female<br>n=0	0 (0.0)	0 (0.0)	0 (0.0)

Notes: VIEU/ml- the unit of serum antibodies titer.

Table 5. Geometric average titer of anti-TBEV antibodies in serum of subjects undergoing complete primary and booster vaccination regarding to sex

TBEV vaccination	Age group	Sex	n	TBEV IgG antibody titer GMT (95% CI) [VIEU/ml]
Complete primary immunization	20-30	Male	72	316 (218-457)
		Female	13	421 (132-1339)
	31-40	Male	158	450 (368-550)
		Female	29	553 (399-767)
	41-50	Male	127	291 (229-371)
		Female	20	451 (262-777)
	51-60	Male	130	252 (197-323)
		Female	31	218 (102-464)
	>60	Male	46	207 (130-330)
		Female	0	-
Complete primary immunization and at least 1 periodic booster	20-30	Male	47	655 (538-799)
		Female	13	860 (677-1093)
	31-40	Male	289	635 (555-726)
		Female	43	689 (548-867)
	41-50	Male	479	613 (563-668)
		Female	48	514 (361-731)
	51-60	Male	492	499 (453-550)
		Female	80	410 (304-551)
	>60	Male	191	517 (452-591)
		Female	7	368 (120-1131)
